# Physically Switchable Antimicrobial Surfaces and Coatings: General Concept and Recent Achievements

**DOI:** 10.3390/nano11113083

**Published:** 2021-11-16

**Authors:** Roman Elashnikov, Pavel Ulbrich, Barbora Vokatá, Vladimíra Svobodová Pavlíčková, Václav Švorčík, Oleksiy Lyutakov, Silvie Rimpelová

**Affiliations:** 1 Department of Solid State Engineering, University of Chemistry and Technology Prague, Technická 3, Prague 6, 166 28 Prague, Czech Republic; roman.elashnikov@vscht.cz (R.E.); vaclav.svorcik@vscht.cz (V.Š.); 2 Department of Biochemistry and Microbiology, University of Chemistry and Technology Prague, Technická 3, Prague 6, 166 28 Prague, Czech Republic; pavel.ulbrich@vscht.cz (P.U.); barbora.vokata@vscht.cz (B.V.); vladimira.pavlickova@vscht.cz (V.S.P.)

**Keywords:** smart nanomaterials, antimicrobial coatings, physical stimuli, smart coatings, antifouling surface, biomedical applications, tailored surface

## Abstract

Bacterial environmental colonization and subsequent biofilm formation on surfaces represents a significant and alarming problem in various fields, ranging from contamination of medical devices up to safe food packaging. Therefore, the development of surfaces resistant to bacterial colonization is a challenging and actively solved task. In this field, the current promising direction is the design and creation of nanostructured smart surfaces with on-demand activated amicrobial protection. Various surface activation methods have been described recently. In this review article, we focused on the “physical” activation of nanostructured surfaces. In the first part of the review, we briefly describe the basic principles and common approaches of external stimulus application and surface activation, including the temperature-, light-, electric- or magnetic-field-based surface triggering, as well as mechanically induced surface antimicrobial protection. In the latter part, the recent achievements in the field of smart antimicrobial surfaces with physical activation are discussed, with special attention on multiresponsive or multifunctional physically activated coatings. In particular, we mainly discussed the multistimuli surface triggering, which ensures a better degree of surface properties control, as well as simultaneous utilization of several strategies for surface protection, based on a principally different mechanism of antimicrobial action. We also mentioned several recent trends, including the development of the to-detect and to-kill hybrid approach, which ensures the surface activation in a right place at a right time.

## 1. Antimicrobial Surface Protection—Global Problem and Related Challenges

Surface bacterial contamination is a relevant problem in a wide range of applications, including medical equipment and implants, protective apparel in hospitals, food packaging, and storage as well as water purification systems [1,2,3]. Associated with bacteria surface colonization and related biofilm formation, the risk of infection is one of the most serious complications in hospital and community settings [4,5]. Bacterial surface colonization commonly occurs through several mechanisms, including hydrophobic and electrostatic interactions [6,7]. Besides, bacterial attachment to a surface often occurs through a previous formation of surface-absorbed protein layers [5,8]. The type of bacteria–surface interaction varies from one bacterial strain to another and can even differ within a particular type of bacteria due to mutations. Therefore, to protect the surface from bacterial attachment, the control of surface hydrophobicity, morphology, or changing the surface charge, as well as surface decoration with antimicrobial agents (or gradual release of these agents from protective coatings), can be considered as a potential way to reduce bacterial attachment or eliminate microorganisms before their adhesion to the surface [9,10,11].

Several strategies for surface protection were developed and experimentally tested, including prevention of bacterial adhesion, killing of adhering bacteria or all surrounding bacteria as well as destroying of actually formed bacterial biofilm [12,13,14]. One simple solution proposed many years before consists in the incorporation of antimicrobial agents in the protective coating structure and achievement of protection against bacterial colonization through a gradual release of these agents or contact killing of bacteria [9,12,14]. However, nowadays, this solution cannot be considered effective because the doped coatings lose their efficiency with time, which represents a high risk for the development of resistant bacterial strains as well as undesired environmental contamination by the released antimicrobials [15,16]. Besides, most of the antibacterial coatings and materials have a similar release profile, which contains a phase with rapid release of antibiotics (sometimes referred to as a burst release) and a second phase with a slow release [17,18]. Commonly, the burst release is not a desired phenomenon because a high concentration of antimicrobial agents can lead to high cytotoxicity of the coating. Finally, when microbes are attached and concentrated on the surface and form a protein layer resulting in the development of a highly treatment-resistant biofilm, this makes the treatment with released or grafted antimicrobial agents ineffective [19,20].

Another alternative strategy is the preparation of antifouling coatings that resist the formation of a bacterial protein layer (responsible for further bacterial colonization of a surface) or bacterial adhesion (these events are usually interrelated, but there are several exceptions) [9,21,22,23,24]. In this regard, immobilization of water-soluble (polyethylene glycol, PEG) or ionic (in common case zwitterionic) polymers has been widely reported [25,26,27]. This approach prevents the formation of the protein-adhesive layer, due to the compression of the polymer chains and the appearance of repulsion from surface elastic forces [28]. Such a method of surface protection is highly efficient, cost-effective, and scalable. On the other hand, surface protection using grafting of water-soluble polymers has several significant and up to now insurmountable drawbacks. First of all, the coated materials have a strong tendency to degrade in the presence of oxygen, high temperature, or sunlight illumination [22]. The effectivity of water-soluble polymer protection depends on the surrounding environment, which makes this surface protection strategy less universal [29]. Finally, the extent of surface bacterial resistance does not linearly correlate with the protein attachment resistance, commonly reported for grafted water-soluble polymers, i.e., the bacteria can colonize the protected surface despite protein attachment resistance [8].

Another strategy of surface protection is based on surface decoration with biocidal molecules, which remain permanently grafted and ensure so-called contact killing [30,31]. This approach does not require the release of antimicrobial agents, keeping their surface concentration at a constant, bacteria-killing level, and does not lead to pollution of the surroundings. Besides, the commonly used antimicrobial agents in contact-killing have a non-antibiotic nature (antibiotics commonly lose their efficiency after changes in their chemical structure during surface grafting)—the quaternary ammonium salts, antimicrobial peptides, and enzyme-based approaches were reported [32,33]. Thus, the contact-killing-based approach is not commonly associated with the risk of antibiotic-resistant bacteria development. Unfortunately, in the case of contact-killing, the accumulation of bacterial residues can inactivate the effect of the antimicrobial agent, also leading to the passivation of surface functionality. Besides, a similar problem, related to the degradation of grafted antimicrobial agents or their gradual leaching, can be expected in this case.

An alternative surface-protection approach is related to the design and grafting of self-cleaning coatings [34]. In this case, the superhydrophobic surface properties should be combined with water repellence to reach a specific, known from nature, “lotus leaf” effect [35,36]. When the water droplets roll down on superhydrophobic surfaces, they pick up bacteria or other (bio)debris, leaving behind a cleaner surface. Such a kind of surface functionality can be achieved through a combination of low surface energy materials and high surface roughness [37,38]. Unfortunately, the creation of a deep surface pattern with the required high height-to-width ratio is not a simple technical task, and additionally, a high risk of mechanical rupture of the coatings can be expected. 

Therefore, many problems remain unsolved in the field of surface protection. Most of them are also related to gradual loss of surface functionality, due to release or degradation of incorporated antimicrobial agents, screening of grafted antimicrobial agents, the low specificity of surface protection, and overall worse control of antimicrobial action [12]. To solve the aforementioned questions, the implementation of a smart approach, aimed at temporarily or spatially separated activation of protective coatings, has been proposed. The main concept of smart surface protection can be described as follows: **protective and antimicrobial coatings should be activated solely at the right time and place to kill or eliminate the bacteria.**

## 2. Smart Coatings—A General Concept

The current research in antimicrobial coating design and realization is focused on the creation of “smart” coatings as intelligent, reliable, long-life, and safe surface protection. Such smart coatings should ensure the delivery of antimicrobial agents only when bacteria are present [39]. These coatings are commonly based on targeted surface decoration with the utilization of so-called smart materials, able to rapidly and reversibly change their physicochemical properties in response to small changes in environmental conditions (for example, chemical conditions—pH, salt concentration, presence of biomolecules, or physical conditions—temperature, electrical potential, light, etc.) [40,41]. Depending on the way of their activation, the smart antibacterial protective coatings can be classified into two groups: chemo or physically responsive ones. In the first case, the coating activation is achieved by the changes in the chemical composition of the surrounding (in an ideal case, associated with bacterial presence detected by a pH change or occurrence of specific biomolecules) [42,43,44]. In the case of physically activated coatings, the application of external triggering by various stimuli (light, temperature, electric, or magnetic fields as well as mechanical stress) is required for activation of antimicrobial properties [45,46]. More detailed information about the chemically activated coating systems (in particular, bacterial-induced) can be found in recent excellent reviews [12,47]. In this review article, we have paid main attention to physically activated coatings.

### 2.1. Temperature-Induced Surface Protection

#### 2.1.1. Surface Antifouling Approach

The class of materials which can undergo temperature-induced phase transition near the “bio”-reasonable range is well known. However, only a part of them can be used for surface decoration, and among them, the temperature-responsive polymers are commonly used [48,49]. These polymers exhibit a lower critical solubility temperature (LCST). Below LCST, they are soluble in water, while above the LCST, they undergo a phase transition to a water-insoluble and collapsed structure [50].

**Figure 1:**
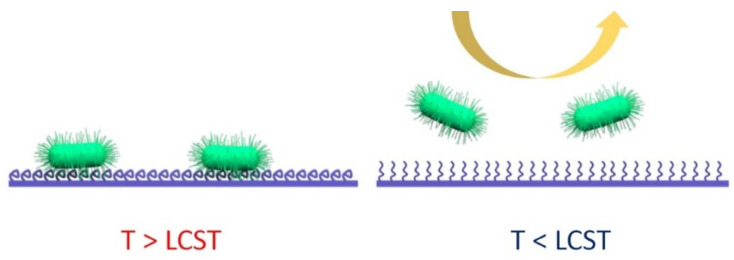
Surface protection using temperature-responsive polymer grafting: bacterial attachment above a lower critical solubility temperature (LCST, the polymer is in a collapsed state; **left**) and bacterial removal below the LCST (the polymer is in a swollen state; **right**).

Thus, by changing the temperature of the underlying material, the (bio)pollutants attached to the thermo-responsive polymer-protected surface can be released, rendering the surface clean (Figure 1). Therefore, thermo-responsive coatings perform a smart antifouling function, which does not damage the bacteria but removes them from the surface on-demand.

Because the thermo-responsive polymers are water-soluble below the LCST, they should be grafted on the surface before sample interaction with water-based media. The surface grafting of polymers can be realized through “surface-from” or “surface-to” approaches. In the first case, usage of the well-known reversible addition-fragmentation chain-transfer polymerization (RAFT) or nitroxide-mediated polymerization (NMP) techniques were reported [51,52,53]. The latter one is based on the utilization of terminated polymer chains (amino-, carboxy- or thiol terminations are commonly used) and their conjugation with the previous grafting of chemical moieties to the surface [54,55,56]. The utilization of both approaches can provide relatively stable grafting of polymer chains, but it is restricted for scale-up reasons because of polymerization conditions or the complexity of terminated polymer chain synthesis. As a solution, the creation of poly(*N*-isopropyl acrylamide) interpenetrating networks with other polymers, or its preparation in partially cross-linked form, can be mentioned [51,52,53,54,55,56]. However, in this case, the “diluted” surface concentration of polymer chains as well as their gradual leaching can lead to worsening antimicrobial surface functionality with time.

The ability of temperature-responsive polymer chains to prevent bacterial adhesion at the temperature below the LCST (and vice versa, the temperature above the LCST supports bacterial adhesion) was demonstrated on *Salmonella typhimurium*, *Bacillus cereus*, and *S. epidermidis* [57,58]. In particular, the detachment of microorganism cells was found to have the reverse character, i.e., surface colonization occurs at a lower temperature, at which the polymer coatings are in a hydrophilic state, while the temperature increase results in removing of the bacteria [48] (Figure 1). In this regard, the coating switching becomes a key point, which justifies their utilization. Unlike the common water-soluble and nonsmart polymers, immobilization of temperature switchable polymers enables to switch the surface properties and remove bacteria independently on the bacterial affinity to surface hydrophilic or hydrophobic states.

#### 2.1.2. Temperature Governed Antimicrobial Release

An alternative way of how to utilize the poly(*N*-isopropyl acrylamide) (PNIPAm) phase transition is related to different diffusion of the incorporated antimicrobial agents in the matrix of partially cross-linked (or mixed with another polymer) PNIPAm [59]. Indeed, at the temperature above the LCST, the polymer chains are “closely packed” with minimal free volume, while coating cooling results in material swelling and free volume increase. As a result, the diffusion and release of incorporated antimicrobial agents are accelerated at temperature below the LCST. A wide range of antimicrobial materials was reported which can be immobilized in thermo-responsive polymers, including silver nanoparticles, antibiotics, or antiseptics as well as antimicrobial peptides [60]. 

The great advantages of thermo-responsive polymer coatings are the ability of reverse trapping and release of antimicrobial agents and coating functionality regeneration because the coatings can be easily recharged by the utilization of temperature-induced phase transition and reversible entrapping/loading of the antimicrobial agent [61]. These types of rechargeable surfaces might find applications in short-term usage because effective recharging requires a high diffusion property of the coating matrix, which in turn results in a faster release of the biocide out of the matrix. 

On the other hand, uncontrolled release of antimicrobial compounds, which is commonly observed even in the coating-insoluble state (above the LCST), can lead to two dramatic consequences: a gradual loss of efficiency as well as a high risk of development of resistant bacterial strains. This drawback is especially topical in the case of antibiotic agents in coatings, where the release of sub-inhibition antibiotic concentrations leads to the development of bacterial resistance with a medically unacceptable degree of probability [62]. Release of other agents, such as antimicrobial nanoparticles or metal ions, can also lead to the occurrence of resistances, as recently reported in ref. [63]. More optimistic results can be expected in the case of antimicrobial peptides or quaternary ammonium salts, for which the development of bacterial resistances was not observed, even though nature knows these compounds for billions of years [64]. On the other hand, syntheses of antimicrobial peptides are not facile; therefore, these compounds will unlikely find an application in the coating of nonspecific, but widely used, “common” surfaces. In turn, quaternary ammonium salts cannot be considered to be chemically stable compounds, especially in the bio-environment and grafted antimicrobial agents thus can lose their functionality with time.

One elegant way, how to overcome the aforementioned limitations is the strong immobilization of grafted nanoparticles or antibiotics to thermo-responsive chains. In this case, bacteria can be eliminated/detached, while the coating is in a swollen state, at which the incorporated antimicrobial agents are open for operation, while the undesired release of the antimicrobial agents can be prevented. Such an approach was reported for silver nanoparticles in ref. [65], but up to now, it was not implemented in the field of smart coatings. Antibiotic grafting to polymer chains can lead to a significant decrease in their activity and careful tailoring of the antibiotic chemical structure is required to avoid this phenomenon [66].

Several alternative approaches for the targeted drug release were also reported on the base of porous drug carriers [67]. The drugs are incorporated inside material pores and the thermo-responsive polymer serves as a valve [68]. At the temperature above the LCST, the pores are plugged by swollen polymer chains and the antimicrobial drug release is blocked. The transition of the polymer to the collapsed state can induce the valve opening and subsequent rapid release of incorporated drugs. To our knowledge, up to now, this approach was not implemented in the field of smart surface protection, but it may provide an interesting alternative, which could make more precisely controlled release of the antimicrobials and, thus, prevent their undesired passive release. 

The main advantages of the utilization of thermo-responsive polymer-based antimicrobial coatings are their well-known chemistry and broad availability. On the other hand, from a general point of view, the implementation of temperature as a driving force for coating activation cannot be considered a universal one. In particular, the temperature stimulus cannot be delivered to the desired places with high precision and accuracy. An additional disadvantage of doped thermo-responsive polymer is related to a so-called burst release discussed above. Another notable drawback for thermo-switchable coatings is their low durability in an aqueous environment because they tend to dissolve in water, leading to a gradual loss of the coating from the substrate [69,70,71]. Therefore, the targeted area of application should be carefully considered for smart thermo-responsive coatings. Nevertheless, thermo-responsive coatings may find a range of advanced applications in both medical and food packaging industries, where the coating durability and a potential burst release are not so critical issues.

### 2.2. Light-Activated Antimicrobial Coatings

Light represents a very inexpensive, common, and renewable source of energy. Thus, utilization of light for activation of antimicrobial properties represents an interesting issue, especially in the case of a large area of outstanding surface coatings, for which sunlight can be used [72]. Additionally, the capability of remote activation, without the necessity of direct contact, can be also advantageous in a range of advanced surface antimicrobial protection applications.

#### 2.2.1. Reactive Oxygen Species-Based Approaches

**Figure 2:**
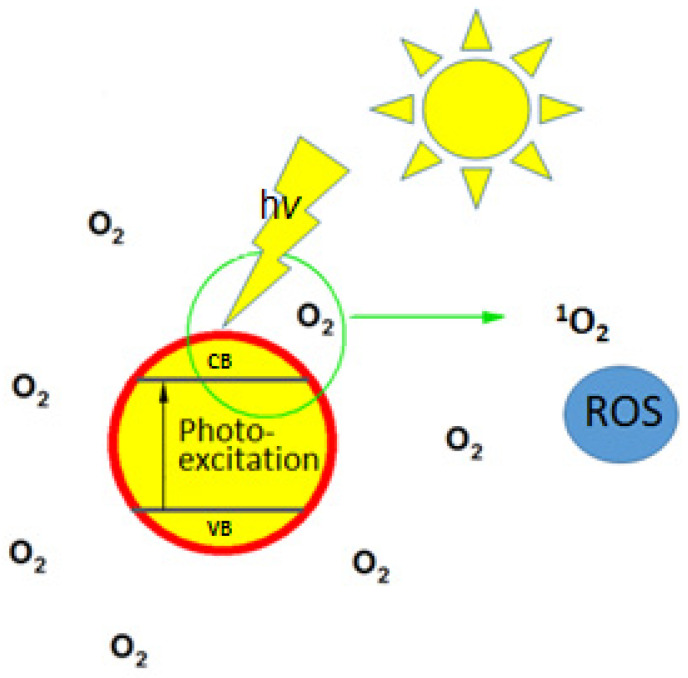
Schematic illustration of light-induced reactive oxygen species (ROS) generation: the creation of singlet oxygen (^1^O_2_) from atmospheric oxygen (O_2_) on the surface of photoactive materials under (sun)light illumination.

In the case of light-activated antimicrobial coatings, the main attention was focused on the addition of photoactive compounds able to produce reactive oxygen species (ROS). The photoexcitation of such coatings and subsequent electron relaxation results in the appearance of superoxide or hydroxyl radicals or energy transferred to oxygen (Figure 2), which is promoted to the excited singlet state [73,74]. The produced ROS can degrade various types of surface contaminants, including absorbed proteins or bacterial cell walls [75].

The advantages of this approach are high efficiency and universality in bacteria-killing. The development of bacterial resistance to ROS cannot be expected in the case of photo-induced treatment with radical production. On the other hand, the high reactivity of radicals can also result in undesired effects because the formed radicals react not only with the target but attack also the surrounding material, leading to its degradation and worsening of mechanical and/or biocompatible properties of the coatings. Thus, incorporation of radical-generated materials should be performed mainly in resistive coating support, such as silicone polymers [76].

Another not fully resolved drawback of the light-activated coatings is the light wavelength, which should be used for radical formation [77,78]. The commonly used TiO_2_ is extremely effective, economically undemanding but has a relatively large forbidden gap, which requires the utilization of UV light illumination. In turn, illumination with high-energy UV photons restricts the material application, due to safety reasons and possible damage of closed biological objects. This drawback can be partially overcome by the preparation of doped TiO_2_, which is active in ROS generation under illumination with visible light [79]. On the other hand, the achieved decrease in the band gap is not sufficient and the utilization of high-energy photons, which can damage biological tissues, is still necessary.

Porphyrins and several dyes (for example, toluidine blue, methylene blue, or Rose bengal [74]) represent an interesting alternative to TiO_2_ because their photoactive antimicrobial effects can be activated by visible light illumination [70,80,81]. These materials exhibit good light-switchable antimicrobial performance in combination with light- and radical-resistant materials, such as medical silicone or polyurethanes [82]. Their light-switchable antimicrobial properties were demonstrated against several bacterial strains such as *E. coli, S. aureus* including its methicillin-resistant strain MRSA, and *B. anthracis* [83,84]. On the other hand, the antimicrobial activity of porphyrins and other dyes may be lost with continued irradiation due to photobleaching and material degradation. The photobleaching of porphyrins and other dyes can be prevented by their coupling to various supports such as metal nanoparticles or carbon-like materials [85,86]. It should also be noted that most of the materials known to produce radicals under light triggering also produce certain amounts of radicals even in the dark. Therefore, their utilization should be restricted by possible damage of mammalian cells and oxidative stress development in the surrounding.

#### 2.2.2. Light-to Heat Conversion

**Figure 3:**
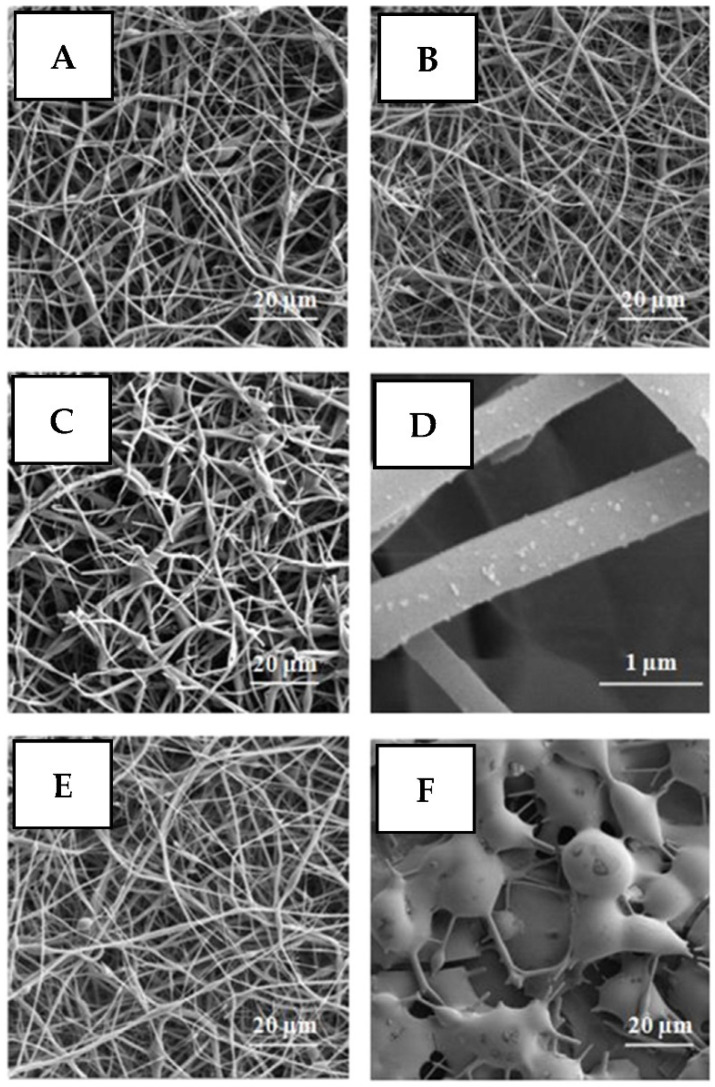
Scanning electron microscopy images of nanofibers prepared by electrospinning from poly(methyl methacrylate) (PMMA) and tetraphenyl porphine (TPP) solutions in chloroform: (**A**) as-prepared Ag/TPP/PMMA nanofibers; (**B**) Ag/TPP/PMMA nanofibers after treatment with light-emitting diode (LED); (**C**) Ag/TPP/PMMA nanofibers after 20-h laser treatment; (**D**) the magnified image of the laser-treated Ag/TPP/PMMA nanofibers; (**E**) TPP/PMMA nanofibers without Ag; (**F**) TPP/PMMA nanofibers without Ag after diode treatment for 20 h. Reprinted with permission from ref. [95]. Copyright 2016 Elsevier.

Additional light activation of coatings’ antimicrobial properties can be performed using the light-to-heat conversion phenomenon [87]. In this case, the energy of absorbed photons is dissipated in the closed surrounding of the absorption center in the heat form, leading to a transformation of the surrounding material(s) [88,89]. Direct utilization of this approach for common light-absorbing materials requires high power of the light illumination for the production of a sufficient amount of heat and material activation in a reasonable time. To overcome this drawback, several strategies can be mentioned, including the decrease in thermodynamic barriers required for heat-induced coatings phase transformation, or light “concentration” near the absorbing surfaces with the utilization of so-called surface plasmon excitation [90,91,92,93,94]. In the first case, the coating material is initially “frozen/prepared” in a thermodynamically unstable state. Thus, even low-intensity light illumination may result in material heating, sufficient for overcoming the residual thermodynamic barrier and coating transformation to a thermodynamically stable state. For example, the polymer fibers doped with light-absorbing silver nanoparticles (which also serve as an antimicrobial agent) can be mentioned [95]. During electrospinning of such fibers, the polymer chains are rapidly conserved in thermodynamically unstable conformation, due to the rapid solvent evaporation. Under the medium power light illumination, the silver nanoparticles heat the surrounding polymer chains, which are transformed into a more convenient conformation. As a result of the chain movement, the silver nanoparticles are extruded from the polymer chains (Figure 3). The proposed approach does not require high power illumination and can be used for on-demand local activation of antimicrobial activity. On the other hand, this method does not enable the reversibility of material transition after light implementation; the material is changed irreversibly and cannot be regenerated through a fiber back-transformation or silver nanoparticle loading.

**Figure 4:**
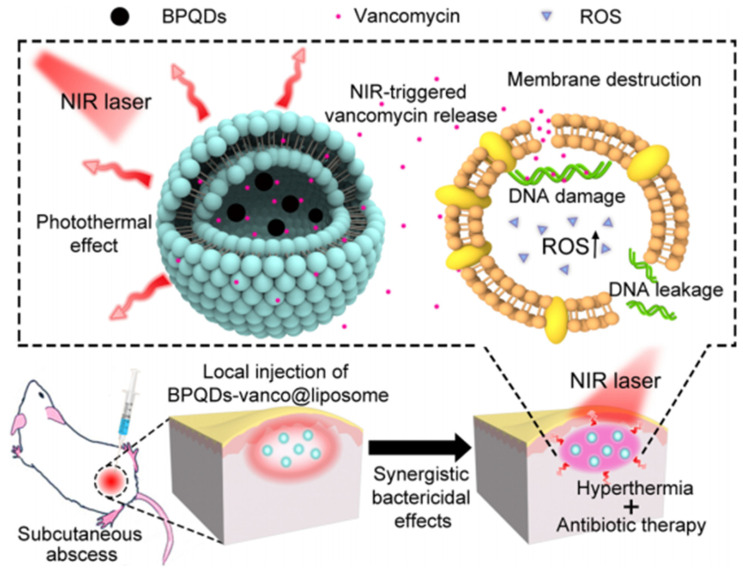
A schematic illustration of the photon-controlled antibacterial platform for the synergistic treatment of a bacteria-infected subcutaneous abscess. Reprinted with permission from ref. [99]. Copyright 2019 American Chemical Society.

An alternative way, how to decrease required illumination power or to shift the required wavelength far from the UV region is the utilization of surface plasmon resonance. The surface plasmon is the collective oscillation of electrons on the (nano)metal-dielectric interface. For surface plasmon excitation, noble metal nanoparticles or nanostructures are commonly being used, and their high photon absorption cross-section provides efficient light energy conversion. Besides, the effective wavelength of the surface plasmon is significantly shorter than the wavelength of incident photons, with the corresponding ability to focus light energy into a very small space (commonly in the range of nanometers), in which an effective light-to-heat transition occurs, leading to a local temperature increase by up to several hundred degrees [96]. The metal nanoparticles can be also conjugated with dyes to extend the wavelength range and to increase the overall light absorption [97]. Such an approach can be used to affect the surrounding material matrix (for example, polymer-based nanostructure) and activate antimicrobial effects, based on antimicrobial agent release or ROS generation (Figure 4) [98,99].

### 2.3. Electrical or Magneto-Responsive Coatings

#### 2.3.1. Magnetic Field

**Figure 5:**
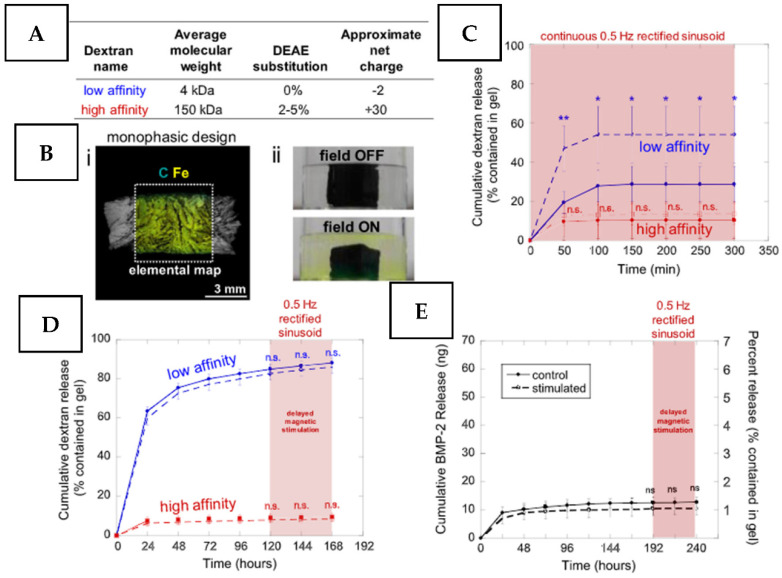
Demonstration of magnetic stimulation for enhanced release from monophasic ferrogels: (**A**) A table providing the theoretical size, diethylaminoethyl substitution, and estimated net charge for the dextrans labeled with fluorescein isothiocyanate (FITC) used in this study. (**B**) (i) Scanning electron microscopy image with elemental mapping of a dehydrated monophasic ferrogel. (ii) Photographs of a monophasic ferrogel loaded with 4 kDa FITC-dextran before (top) and during (bottom) magnetic compression. (**C**) Cumulative release vs. time for low (blue) and high (red) affinity dextrans when monophasic ferrogels were either not magnetically stimulated (solid) or continuously stimulated with a 0.5 Hz rectified magnetic gradient (dashed). (**D**) Cumulative release vs. time for low (blue) and high (red) affinity dextrans when monophasic ferrogels were either nonmagnetically stimulated (solid) or stimulated with a 0.5 Hz rectified magnetic gradient from 120 to 168 h (dashed). (**E**) Cumulative and percentual release of mouse bone morphogenetic protein-2 (left and right vertical axes, respectively) vs. time when monophasic ferrogels were either not stimulated (solid) or stimulated with a 0.5 Hz rectified magnetic gradient from 192 to 240 h (dashed). In parts C–E, statistics were applied to compare the stimulated release to unstimulated release (N¼4). Reprinted with permission from [108]. Copyright 2018 Elsevier.

The magneto-based control of coating behavior represents an elegant approach because magnetic fields can be easily generated and controlled with the advanced capability of removed antimicrobial material activation and low risk of surrounding tissue or material damage [100,101]. Mainly, the design of magneto-responsive coatings supposes their doping with antimicrobial agents and magnetic nano- or micro-particles [102,103]. In this case, the significant acceleration of the drug release in the presence of a magnetic field was reported [104]. The magneto-triggered antimicrobial release is attributed to increased permeability of the coating under external field application [105,106]. However, the increase in permeability can lead to mechanical or chemical rupture of the coating or its heating under the application of periodical triggering [100,107]. The amount and kinetic of magnetic field-triggered drug release can be significantly tuned using the field strength and frequency [104]. In particular, the optimization of a coating triggering mode and the introduction of more complex biphasic stimuli application (Figure 5) enables reaching excellent control of the incorporated drug release [108]. Another interesting example of the ability of magneto-controlled directed motion for enzyme-based antimicrobial regeneration was recently reported [109].

**Figure 6:**
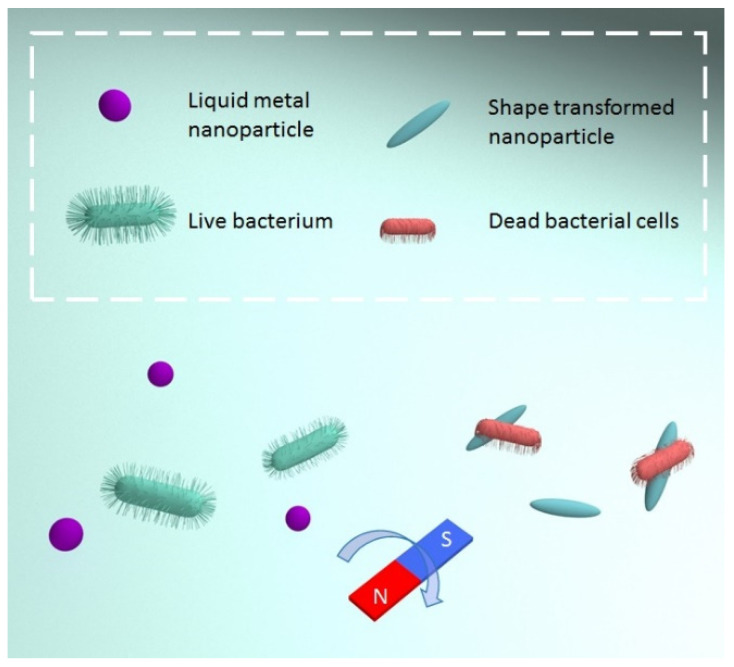
A schematic illustration of the actuated gallium-based liquid metal (GLM-Fe) particles. (A) Shape-transformation of the actuated GLM-Fe particles from spherical to high-aspect-ratio shapes when exposed to a rotating magnetic field.

A very interesting example is the recently reported utilization of magneto-responsive nanoparticles with the ability of sharp transition under magnetic field application (Figure 6). Reported nanoparticles, based on gallium liquid metal, were able to induce mechanical disintegration of microorganisms and biofilms [110,111]. In particular, the appearance of shaped edges on nanoparticles under magnetic field application and magneto-induced nanoparticle motion destroys the bacterial biofilm and physically ruptures the bacterial cells with the dense biofilm matrix being finally broken down. Such an approach can work on the entire scale of microorganisms, and the risk of resistant strain development can be ignored [16]. The method was reported for non-immobilized nanoparticle suspensions, but the grafting of nanoparticles on a protected surface seems to be a very interesting alternative. Similarly, magneto-responsive coatings, able to change morphological features under external magnetic fields, are widely used in the field of tissue engineering to control mammalian cell detachment or govern their behavior [112,113,114].

According to the recent works, the application of magnetic stimuli can find more wide and interesting applications in the area of surface protection because it can provide an opportunity to reversibly change the surface morphology and wettability, with related ability to tune the surface free energy and proteins or bacterial adhesion. Reversible changes from “flat” to “sharped” surface may on demand introduce the sharped surface feature able to damage and kill the attached bacteria. Such magneto-responsive coatings can be easily realized through matrix coating doped with magnetic nanoparticles. Simpler and scalable techniques, such as in situ crystallization and freeze-drying, co-precipitation, molding, and solvent-casting techniques [115,116,117,118,119] provide an elegant strategy for the creation of smart, magneto-activated coatings. Subsequent utilization of the external magnetic field results in significant surface morphology changing, providing an opportunity to create and switch proteins adhesivity or recreate dynamically cell-repellent surfaces.

**Figure 7:**
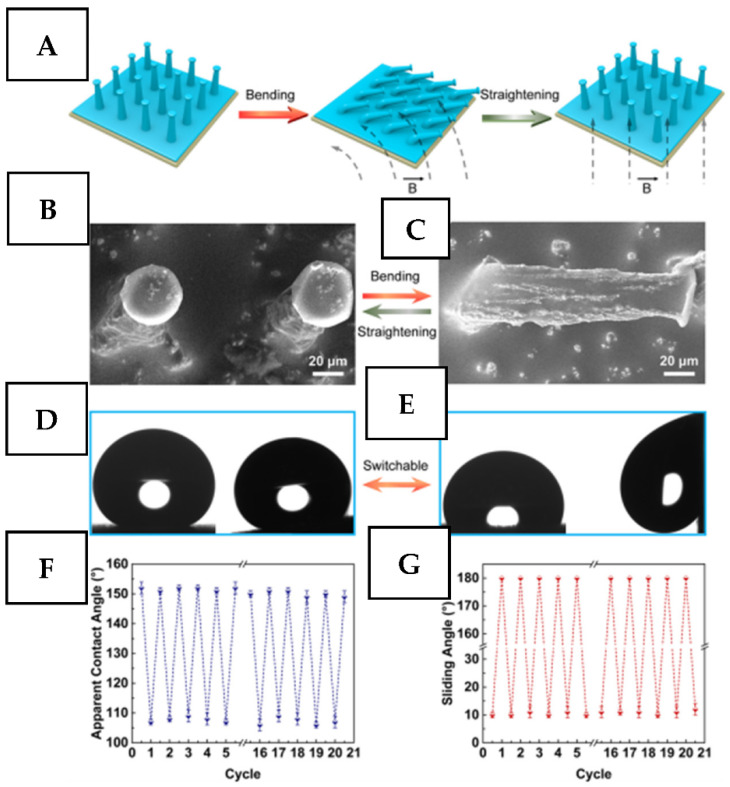
(**A**) A scheme of the reversible shape transformation between the upright state and the nearly flattened state. (**B**,**C**) Scanning electron microscopy images of the mushroom-like pillars in the upright and curved states, respectively. (**D**,**E**) Switchable wettability of the surfaces for hexadecane corresponding to (**B**,**C**), respectively. (**F**,**G**) Reversible variations in the apparent contact angle and sliding angle of hexadecane through repeated bending and recovery processes, respectively. Reprinted with permission from ref. [120]. Copyright 2019 American Chemical Society.

**Figure 8:**
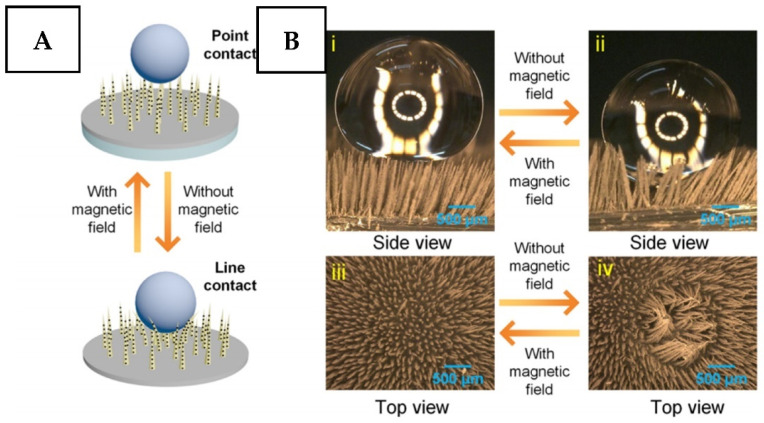
(**A**) A scheme for reversible switching of wettability and adhesion properties of the magnetically responsive superhydrophobic surface by on/off switching of the magnetic field. (**B**) Optical microscope images show the stiffness tunability of the magnetorheological elastomer micropillars under an external magnetic field, which can transform the micropillars from the collapsed morphology (water-adhesive state) to the fully upright position (water-repellent state). Reprinted with permission from ref. [121]. Copyright 2018 American Chemical Society.

The reversible tuning of surface morphology from rough to flat one under the application of the magnetic field was described in ref. [120,121] and is demonstrated in related Figure 7 and Figure 8. Such an approach was used for reversible switching of surface water and oil wettability, but a similar approach can be also used for active removal or disruption of adhered surface protein layers or bacteria. A similar approach was also described in ref. [122], in which the authors used the magnetic field to control surface morphology and dynamic wettability of the samples prepared by deposition of magnetic Fe_3_O_4_ nanoparticles and subsequent coverage with a silicone polymer. The main attention in this field was focused on achieving the switching between ultimately opposite surface states (superhydrophobic/superhydrophilic or water adhesive/water repellent) [123,124,125]. Such surfaces can be utilized in the flow regime in an initially flat state, without the risk of damage of the morphology features. For the protection against bacterial colonization, the surface can be easily converted into a highly roughed state with related repellence of an absorbing layer, protein layer, or bacteria. Besides, due to the simplicity of magnetic field introduction, the surface activation can be easily performed in several subsequent cycles, without any chance of bacteria remaining attached to the protected surface. Therefore, the emergence of new works in which magneto-responsive surface coatings will be used for on-demand activated surface protection can be expected shortly.

#### 2.3.2. Electro-Responsive Coatings

**Figure 9:**
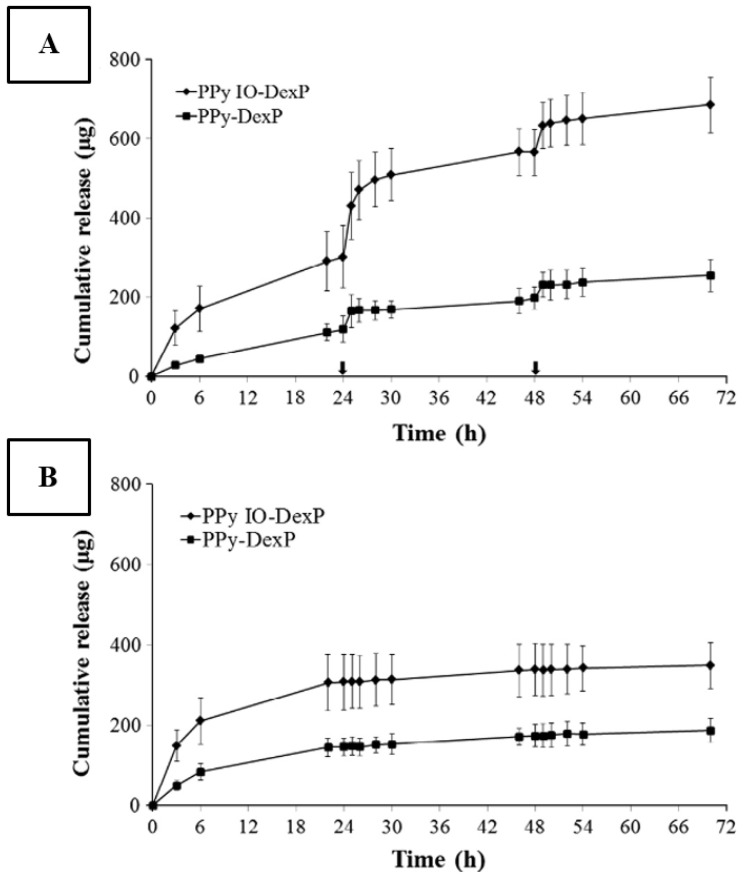
(**A**) Drug release profiles for polypyrrole inverse-opal dexamethasone phosphate films and conventional nonporous polypyrrole dexamethasone phosphate films. Following 24 h of a passive release, 1 h periods of electrical stimulation were applied (arrowed time points) at 24 and 48 h (*n* = 3, mean ± standard error) and (**B**) control drug release profiles, without electrical stimulation, for PPy IO–DexP and conventional PPy–DexP films (*n* = 3, mean ± standard error). Using electro-responsive macroporous polypyrrole scaffolds for triggered dexamethasone delivery. Reprinted with permission from ref. [128]. Copyright 2015 Elsevier.

The common approach in the field of electro-controlled smart antimicrobial surfaces is based on the formation of porous or high-surface-area materials able to reversibly entrap/release the antimicrobial agents through electro-static interactions [126,127]. An additional requirement for such material is the presence of redox-active atoms, on which the charged antimicrobial agents can be bound. In this regard, conductive polymer coatings or polymer composites with redox-active nanomaterials are widely used [128,129,130,131]. Charging/discharging of conductive polymers (for example, polypyrrole; Figure 9) makes possible the reversible entrapping/release of a drug or release of antimicrobial agents even in a stepwise matter or pulsatile manner [132].

**Figure 10:**
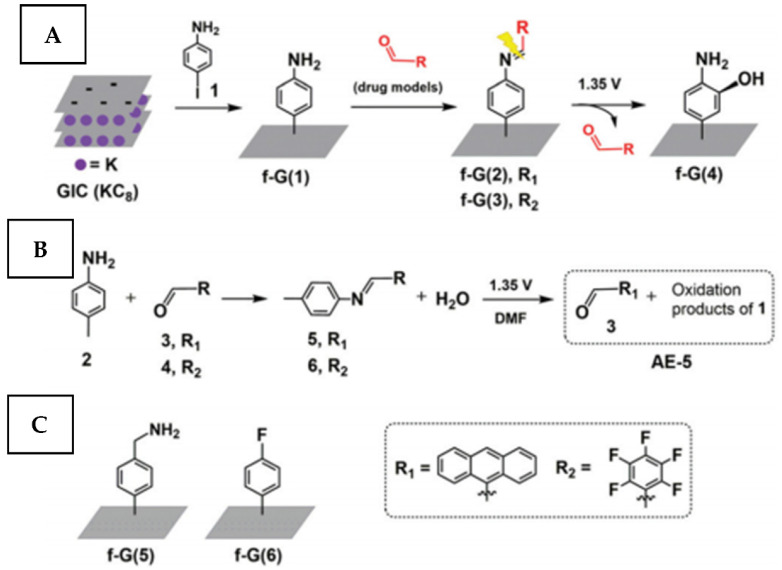
A scheme of the preparation of (**A**) a graphene-based platform f-G(1) loaded with drug models (f-G(2–3)) followed by their electrochemically triggered release (f-G(4)); (**B**) graphene-free organic models employed to characterize the release mechanism; (**C**) graphene-based materials for control experiments. Reprinted from ref. [137] with permission from the Royal Society of Chemistry.

The proposed approach is especially interesting because it does not require the chemical grafting of antimicrobial agents, maintaining the material in a “pristine” state, i.e., with the previously optimized chemical structure for maximal antimicrobial effectivity. This strategy of surface protection is well compatible with metallic-based conductive implants [133], for which the possibility to control the released drug kinetic by the applied voltage is highly desired [134]. On the other hand, the method is limited by requirements for substrate conductivity and the number of charged antimicrobial agents, which implements the utilization of antibiotics as immobilized antimicrobials [135]. Besides, the passive release of immobilized drugs is a common problem due to the weakness of electrostatic interaction [136]. As an alternative approach, the formation of more stable covalent bonds, degradable by electrolysis (Figure 10) was recently proposed [137]. In this case, suppression of undesired spontaneous release can be expected.

Finally, it should be mentioned that most of the organic polymers, commonly used for electric field-driven drug entrapping/release, are not stable against repeated cycles or oxidation/reduction, and, thus, the real coating regeneration should be attributed to conductive polymer stability (100× cycles).

**Figure 11:**
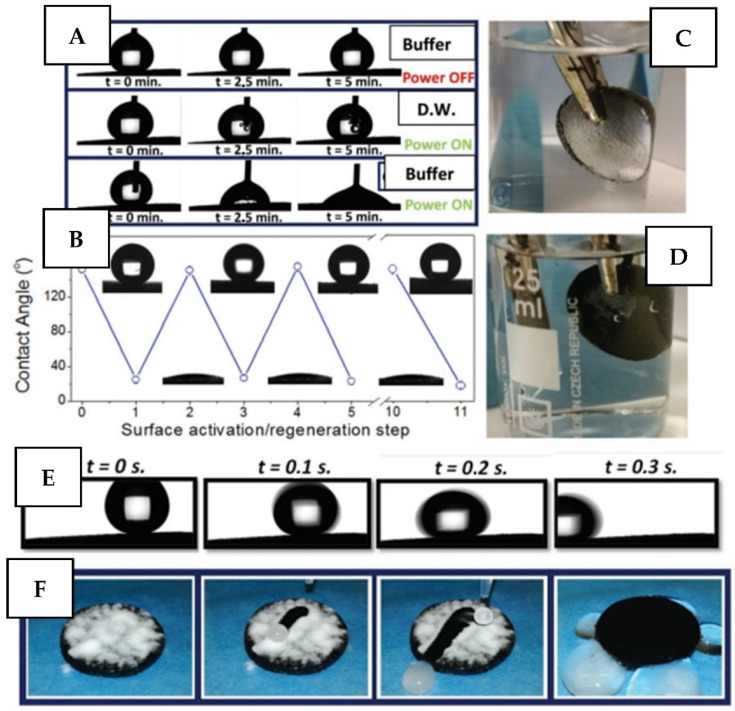
Advanced functionalities and electric field (EF) switchable behavior of polypyrrole functionalized silicon surface: (**A**) Time-dependent behavior of water droplets (deionized water or ions containing water) on the sample surface with and without application of EF; (**B**) repeated cycles (EF on/off) of electro-induced surface transition from a superhydrophobic state to a hydrophilic one (surface activation/regeneration); (**C**,**D**) photography of the samples immersed in water before and after application of EF triggering; (**E**) time-dependent water droplets slipping along the slightly tilted (5°) sample surface; (**F**) example of self-cleaning test on the pristine (or regenerated) sample surface. Reprinted with permission from ref. [142]. Copyright 2019 Wiley.

Another interesting application of electric field-based coating triggering is likely with magnetic field application related to the reversible changing of surface-liquid interaction. In this regard, the well-known electrowetting phenomenon is commonly used [138], where the electric field-induced water precipitation/removal in surface pores or valleys enables the liquid contact angle tuning [139]. As a result, the precise control of the surface wetting is achieved, with related on-demand introduction of superhydrophobicity or water repellence. With such surface designs, smart activation of surface self-cleaning and bacterial repulsion functionality can be achieved [140,141,142]. In ref. [142] a switchable material based on highly stretchable silicone polymer doped with polypyrrole was proposed. The electro-controlled surface self-cleaning performance enables on-demand removal of adhered bacteria or other biocontaminants (Figure 11).

**Figure 12:**
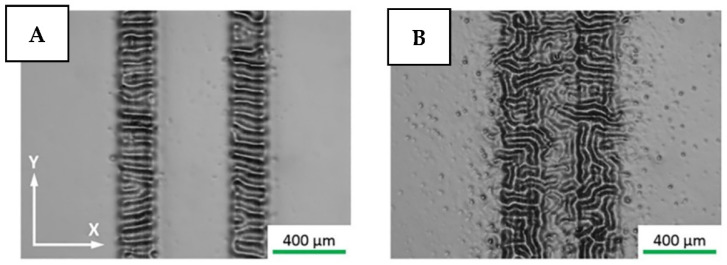
Optical microscopy images of spatially selective and reversible surface pattering by electrode printing—impact of the distance between two electrode lines: (**A**) 400 mm, and (**B**) 200 mm. Reprinted with permission from ref. [148]. Copyright 2020 Elsevier.

The changes of surface wettability under an external electric field can be also realized using a reversible modification of the surface topography [143,144]. In particular, the tuning of flexible film morphology can be achieved by the application of a perpendicular electric field, which induces the surface hydrodynamic instability and wrinkled pattern appearance [145,146,147]. In the common case, this phenomenon was known to be irreversible, but recently the reversible and spatially selective surface morphology tuning was demonstrated, see Figure 12 [148].

**Figure 13:**
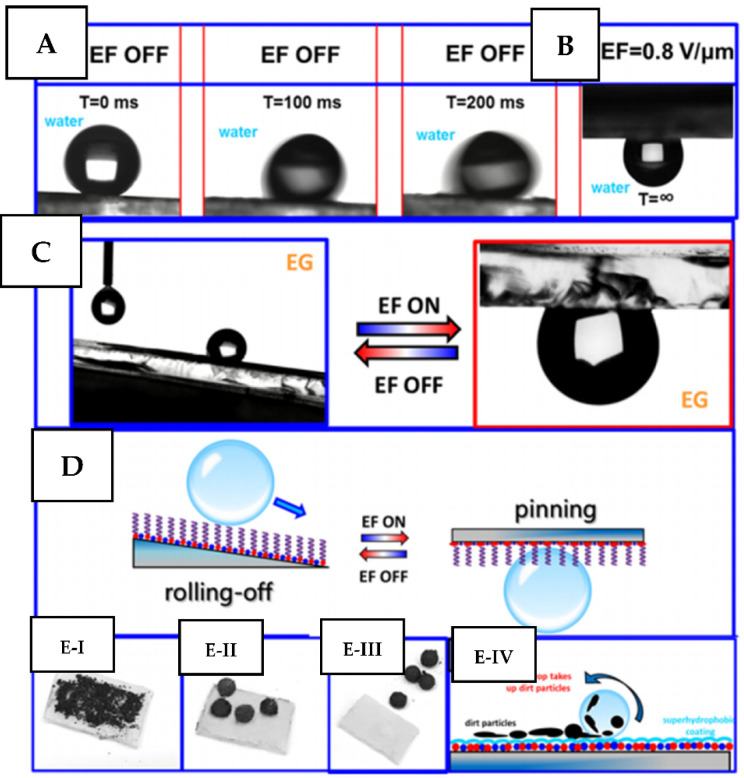
Superoleophobicity and reversible water and ethylene glycol adhesion switching on −C_6_H4−C_8_F_17_ grafted polyvinylidene fluoride/poly(methyl methacrylate) (PVDF/PMMA) fibers: (**A**) a time-resolved image of a 5-µL water droplet at a roll-off angle of 4° without the electric field, (**B**) a resolved image of a 5-µL water droplet at 180° with an EF application; (**C**) optical images of reversible EG adhesion under EF switching on/off; (**D**) a schematic illustration of EF-induced liquid adhesion switching; (**EI**) coating of fibers with graphite powder, (**EII**) application of water a droplet and adsorption of graphite on the droplet after its movement, (**EIII**) cleaning of the surface after sliding off the surface, (**EIV**) a proposed mechanism of the self-cleaning process (From [151]). Copyright © 2018 American Chemical Society.

As an alternative approach, the utilization of electric field can be used for surface geometry switching using the piezo effect [55,149,150,151,152]. In particular, the grafting of piezo-responsive PVDF/PMMA polymer fibers with fluorinated chemical moieties was reported to produce the intrinsically hydrophobic surface. Subsequent utilization of electric field triggering enables us to switch the surface between water repellent/water adhesive states and reach the smart, externally controlled self-cleaning phenomena (Figure 13).

**Figure 14:**
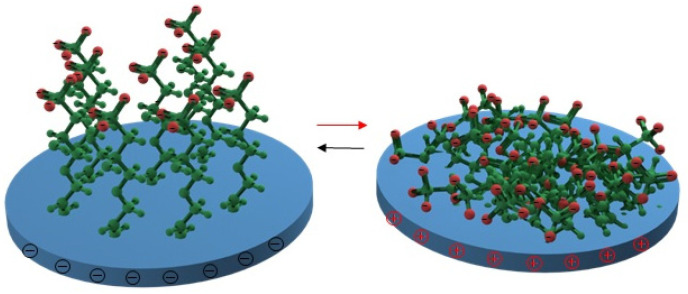
A schematic molecular model of the stretched and rolled electro-controllable poly-sodium allyloxy hydroxypropyl sulfonate-branched brushes after being negatively and positively charged [123].

In the area of the electric field-driven surface protection, attention also deserves changing of the conformation of polymer brushes, grafted to the conductive surface, through its reversible positive or negative charging [123]. In ref. [123], the authors used the proposed approach for electro-switchable friction control, but the detachment of a protein layer or bacteria seems to be an interesting alternative in this case (Figure 14). The switching was reached with the utilization of poly-sodium allyloxy hydroxypropyl sulfonate; however, a wide range of zwitterion polymers can potentially produce similar effects under electric field triggering [153].

### 2.4. Mechanically Responsive Coatings

The mechanically driven antimicrobial protection of the surface can be considered as a perspective avenue in the field of coatings that are exposed to periodical strain due to human action. For example, daily human motion provides a constant source of mechanical stimuli for promoting drug release in a spatiotemporal manner. Additional examples of mechanically responsive coating applications can be related to handrails in public places. Thus, the design and creation of mechanically activated antimicrobial coatings seem to be interesting in both external and in vitro applications.

In the case of mechanical-based antimicrobial surface protection, the specially designed surface morphology with sharp features, like TiO_2_ nanopillars or black silicon surface, is often considered [154,155,156]. The sharp surface features induce the damage of the bacterial cell wall and related bacterial lysis. Recently, it was demonstrated that the nanopillar array can mediate oxidative stress, also ensuring the protection of an antimicrobial surface [155]. Such a strategy cannot be intrinsically considered a smart one because the surface morphology in common sense cannot be switched. On the other hand, the effect of mechanical damage of bacteria can be combined with other treatments, for example, photoactivation of TiO_2_-producing reactive oxygen [157]. An additional interesting example is a combination of superhydrophobic surface and mechanical bactericidal surface properties, not only killing the bacteria but also removing the bacterial residues [158].

**Figure 15:**
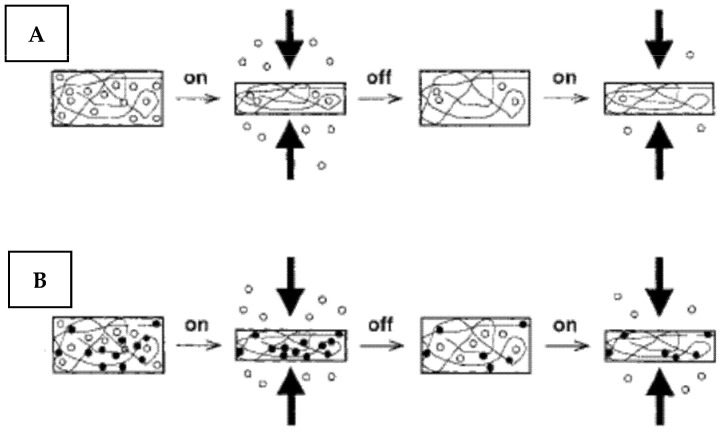
A scheme of anticipated drug release from polymeric matrices under mechanical signaling. (**A**) Expected release behavior of the drug that does not strongly interact with the matrix. (**B**) Proposed release behavior of the drug that interacts with the matrix (white circle—free drug; black circle—bound drug). Reprinted with permission from ref. [163]. Copyright 2012 Wiley.

The widely used way of coating or material mechanical activation is related to drug incorporation in the structure of elastic coatings with a subsequent release under mechanical stress [159,160]. The drug release can be induced by different mechanical triggering, including the application of compression forces, material stretching, or bending (Figure 15) [161,162]. In all cases, the drug release kinetic depends on its binding to the elastic support [163,164]

Indeed, a mechanically responsive coating design and the kind of incorporated antimicrobial agent should be created concerning its further application. For example, the release under mild coating compression can be considered as a protective coating for handrails in public places [165,166]. Oppositely, the high mechanical tolerance should be rather compatible with utilization involving large deformations, like wearable coatings. 

The mechanically responsive approach, however, has a significant drawback—most of the incorporated drugs cannot be bound to the coating structure, and, thus, burst or passive release is inevitable. Thus, for the improvement of release performance and achievement of better control, the special coating matrix design is often required [167,168]. An alternative approach was proposed based on piezoelectric materials, where the application of mechanical triggering led to bacterial degradation due to surface potential generation on the material surface [169,170]. The harvesting of mechanical energy and its conversion to electro-chemical potential led to the production of ROS, ensuring bacterial cell disruption [171]. Such kind of material protection was demonstrated using the BaTiO_3_ material, which is known for its high piezoelectric coefficient, but recently was also reported for polymer films with lower piezo-coefficient [172].

### 2.5. Mixed Approaches

In this part of the review, we describe the recent progress in the extremely interesting and attractive field of multifunctional coatings, including double or multiresponsive coating activation, a combination of bacteria detection and release, killing and removal, as well as simultaneous bacteria deactivation using the mixed mechanism of antimicrobial action [173,174].

#### 2.5.1. Physico-Chemical Stimulation

The introduction of coating multiresponsivity towards two or more external stimuli makes possible better control of coating response in terms of activation of antimicrobial properties. The applied stimuli can combine the physico-physical or physico-chemical options of coating material activation. 

In the case of physico-chemical activation of coatings, the most widely used chemical stimulus is pH [175]. The combination of pH and temperature responsivity was reported many times for the on-demand release of traditional antibiotics, metal ions, or nanoparticles as well as antimicrobial peptides [176,177]. Alternatively, the release from chitosan-based thin films was demonstrated as a function of pH and applied electric potential [178,179,180]. Reported electro- and pH-coating activation are especially interesting in the case of implant protection, taking into account that in patient body implants will be subjected to constant pH (different from “initial”) and activated by electro-triggering. Since not all implants are conductive, the alternative utilization of magnetic field and pH activation proposed in ref. [181] can be also considered as the very perspective way. Finally, the combination of other stimuli, like light and supramolecular host-guest chemical interaction, was also reported [182], and further progress in this field can be expected.

It should be also noted that the combination of physical and chemical sensitivity can potentially open a very interesting capability of local activation of a coating at the right bacteria-colonized place. In this case, an ideal physically chemical-sensitive coating will activate external triggering solely in the place of bacteria colonization. Realization of this ambitious task will enable us to reach two main goals: prevent the incorporated drug unnecessary losses due to triggering of uncolonized places and simultaneously protect the coating from false activation during its storage.

#### 2.5.2. Physico-Physical Stimuli Combination

In the case of two physical stimulus-based coating activations, the most common way of material design is the combination of electro-switchable incorporated drug release with thermo-switchable drug penetration into the surrounding. Such an approach can be realized with composite materials or with copolymers, where the kinetic of drug release is controlled remotely by the application of one or both stimuli [183]. As a typical example, the thermo- and electro-responsive material were designed on the base of chitosan/pluronic/polyaniline loaded with antimicrobial agents [184]. Utilization of thermo-switchable coating matrix and electro-driven drug release additionally enables a double surface protection—controlled drug release and bacteria residue detachment, ensured by thermo-responsive material parts [185]. 

Another attractive way of physico-physical coating activation is the implementation of non-invasive stimuli, such as light and magnetic fields. As an example, magneto- and photo-responsive materials based on material, which contained light-responsive, magneto-responsive, and drug chelating units were reported [186]. An alternative combination of magnetic and mechanical stress for surface morphology tuning was reported in [120]. The authors used a very simple and scalable approach for surface pattering, based on the mix of magnetic particles with silicone resin precursor and film curing under a constant magnetic field. Taking into account the shape and width/height ratio of the created pillar array, the proposed approach represents an interesting way how to create the coatings, which can on-demand prevent bacterial attachment or remove their residues. Other various combinations were also reported in the literature, including temperature, and more rarely used ultra-sound or mechanical triggering [187,188].

#### 2.5.3. Multiresponsive Coatings

**Figure 16:**
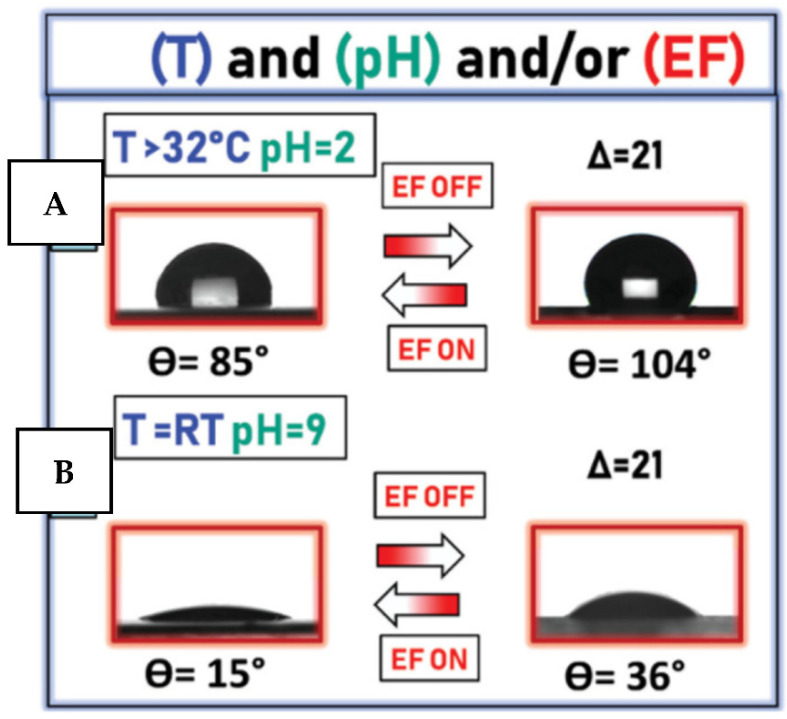
Cyclic application of EF (0.8 V·µm^−1^) results. The surface is triggered in two different wettability states, introduced by polyacrylic acid and poly(*N*-isopropyl acrylamide): (**A**) surface is hydrophobic (pH = 2, T = 35 °C), (**B**) surface is hydrophilic (pH = 9; room temperature). Reprinted with permission from ref. [150]. Copyright 2012 Wiley.

The utilization of more than two ways of coating activations was also commonly considered for smart surface preparation, with potential antimicrobial functionality. For example, in ref. [150], the surface wettability and morphology were found to be affected by the simultaneous triggering by pH, temperature, and electric field. To reach such multi-responsivity, the combination of PNIPAm/PAA polymers was used (Figure 16). PNIPAm and PAA provide the responsivity towards temperature and pH, respectively, while PVDR/PMMA supports the sensitivity towards the external electric field. In this case, the main question, however, is to find some gold standard between the coating complexity, related technological demands, and the degree of surface properties control.

#### 2.5.4. Multidrug Approach

The coating’s multifunctionality can be also reached through the introduction of several mechanisms of agents release or antimicrobial action. In the simplest case, the multidrug loading with different release rates can be mentioned [189,190,191], ensuring temporally stable surface protection. Another interesting and very promising idea is the incorporation of two antimicrobial agents, desirable with different mechanisms of action [192,193]. The combination of silver nanoparticles and antibiotics can be considered for such coating design because of their synergic action (silver nanoparticles damage bacterial cell walls and facilitate penetration of antibiotics) [194]. In another work, Li et al. [195] fabricated antimicrobial materials with dual-function based on silver ions or nanoparticles and immobilized quaternary ammonium salts (QAS). An interesting combination is a quaternary ammonium salt with a photosensitizer chlorin e6 so that the chemical and photodynamic therapy are merged in one moiety for a more efficient antibacterial activity [196]. ROS generation in combination with silver nanoparticle-based bacteria-killing is another simple but effective alternative [97,197]. Indeed, the incorporation of several bacteria-killing approaches in the framework of single coatings can significantly decrease the risk of resistant strain development or kill the previously developed resistant strains [198]. Moreover, the synergy in antimicrobial action is often observed [199], ensuring a high degree of antimicrobial activity, highly desired in specific medical applications (for example, implants surface protection) [200].

#### 2.5.5. Killing and Release

**Figure 17:**
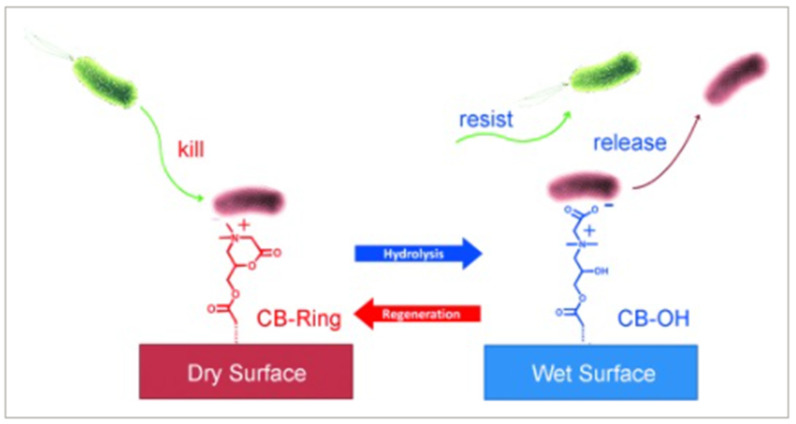
A smart polymer coating repeatedly switches between the attacking function (cationic ring (CB-ring), to kill bacteria under dry conditions) and defending function (zwitterionic carboxy betaine (CB-OH), to release and resist bacteria under wet conditions). CB-Ring can be hydrolyzed to CB-OH in neutral or basic aqueous solutions and can be regenerated by dipping CB-OH in acidic media. Reprinted with permission from ref. [203]. Copyright 2012 Wiley.

An extremely effective approach for the development of smart and very efficient antimicrobial coatings is the creation of self-cleaning and bacteria-killing surfaces [29,141,201,202]. When a released antimicrobial agent kills bacteria, there is a high chance that bacterial residues will contaminate the surface, thus leading to the passivation of its antimicrobial properties. To prevent this undesired scenario, the coating should combine antimicrobial and antifouling properties [203,204,205], like in the case presented in Figure 17. Surfaces and materials with bactericidal and fouling release functions can be tailored in a switchable manner, enabling us to reach strong biocidal effects and on-demand release of dead cells [206,207]. In this regard, several combinations were proposed [208,209], including the release of silver nanoparticles for bacteria-killing and surface grafting with zwitterionic polymers for the introduction of the antifouling performance. An alternative example was demonstrated using the hybrid poly(*N*-hydroxyethyl acrylamide)/salicylate hydrogel doped with silver ions [210].

**Figure 18:**
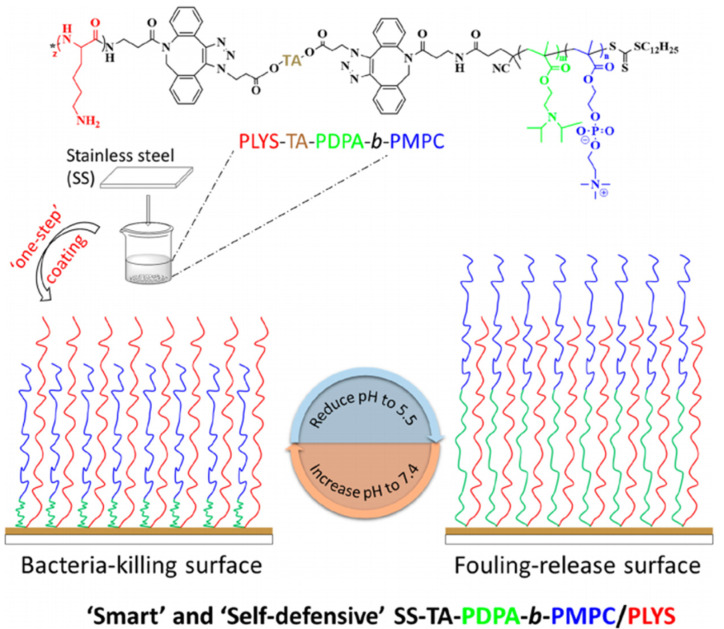
Chemical structure of tannic acid (TA)-scaffolded polymer and a schematic illustration of “one-step” deposition for “smart” and self-defensive TA-scaffolded binary polymer brushes coating. Reprinted with permission from [211]. Copyright 2020 American Chemical Society.

Especially interesting is surface immobilization of both antimicrobial and antifouling materials on the protected surface to reach the contact killing and removing of killed bacteria. Such an approach was reported in ref. [203], in which the polymer surface switching between a cationic and zwitterionic state enables bacteria-killing and residue removal. Similar switching from a cationic surface to a zwitterionic surface was reported in ref. [29], leading to the elimination of the bacteria and preventing bacterial contamination by the remnant residues. Similarly, in ref. [211], the external switching of the surface enables on-demand activation of the antimicrobial or antifouling surface functionalities (Figure 18). In the reported cases, the antimicrobial surface can be also engineered using immobilization which undergoes reversible chemical transformation for bacteria-killing or removal [29,203]. Such a kill-and-remove approach may overcome the principal mismatch in a coating design and action: antimicrobial materials have to interact with microbes while antifouling materials tend to repel them. The special design of grafted molecules may achieve a potential compromise by adjusting the temporal presence of the two materials through external stimuli, enabling only one type of material to appear and function at a proper moment.

**Figure 19:**
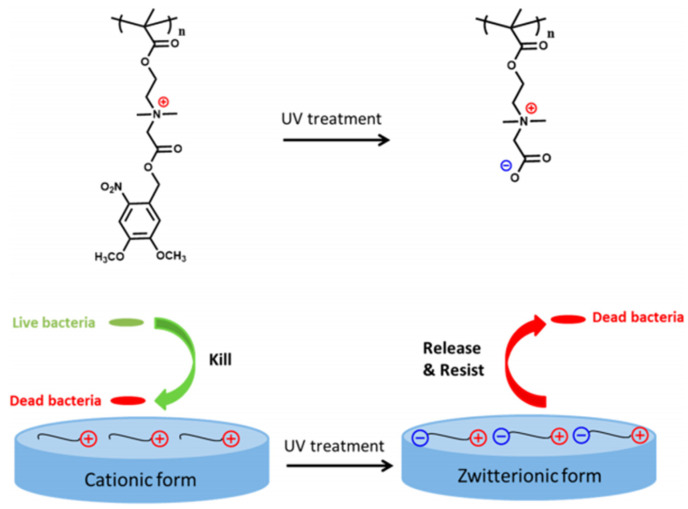
A schematic illustration of poly[2-((4,5-dimethoxy-2-nitrobenzyl)oxy)-N-(2-(methacryloyloxy)ethyl)-*N,N*-dimethyl-2-oxoethan-1-aminium] hydrogel surfaces switching from cationic antimicrobial to zwitterionic antifouling form upon UV photolysis. Reprinted with permission from ref. [212]. Copyright 2019 American Chemical Society.

Additional interesting examples including the light-switchable hydrogel-based coating were reported [212]. In the proposed design, the photolabile groups can be cleaved from the hydrogel shortly upon UV irradiation and the polymer surface switching from an antimicrobial cationic to a repellent zwitterionic form can be achieved (Figure 19). The cationic hydrogel as a precursor was shown to effectively kill the attached bacteria, and then it can be quickly switched via photolysis to zwitterionic antifouling form, releasing the attached bacteria from the surface and preventing further bacterial attachment.

**Figure 20:**
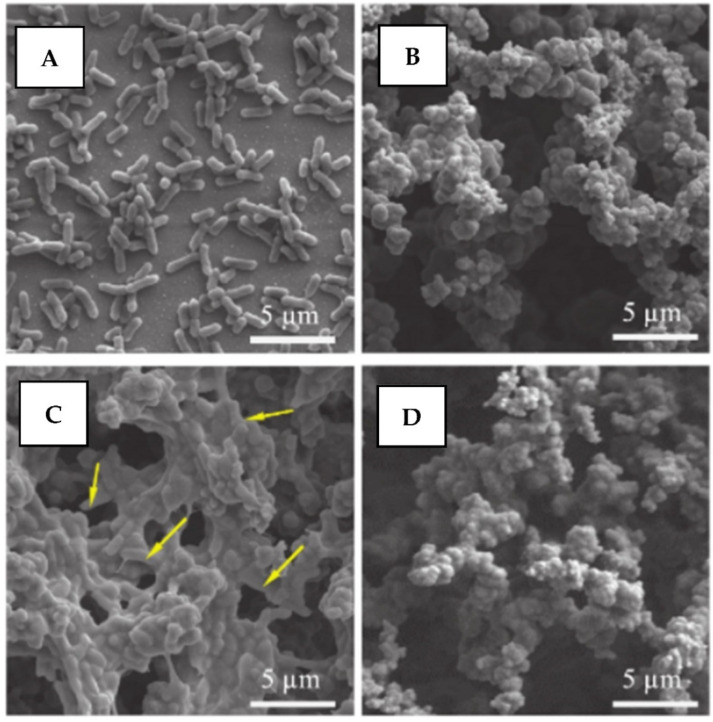
Scanning electron microscopy images of silicone/polypyrrole sample surface after the interaction with *E. coli* for 15 h: (**A**) control sample (Au covered with a thin copper film); (**B**) without and (**C**) with the application of EF; (**D**) *E. coli* contaminated sample surface (the same as in (**C**)) after simple rinsing with water. Reprinted with permission from ref. [142]. Copyright 2019 Wiley.

Finally, an alternative approach was proposed in ref. [142], in which the material, based on the flexible silicone polymer doped with conductive polypyrrole, was electrochemically loaded with the antimicrobial agent. In its pristine state, the proposed polypyrrole layer demonstrates the superhydrophobic and self-cleaning behavior. The application of electric field triggering can convert the material into a hydrophilic state and induce the simultaneous release of the incorporated antimicrobial agents (Figure 20). As can be expected, the bacteria at the protected surface are killed and their residues contaminate the surface. However, the electric field switches off the return of the surface to the initial superhydrophobic and water adhesive state, making the bacteria residues removal possible by simple washing.

#### 2.5.6. Detect and Kill

In all cases of physical stimulus application, one dubious question remains: At what time should be the stimuli applied to activate the antimicrobial properties? From this point of view, the creation of multifunctional surface coatings, which combines bacteria-sensitive and on-demand activated antimicrobial properties, seems to be a prospective avenue for further research. In this case, the first external “back” stimulus means that an infection was detected (for example, through monitoring of physiological parameters), and only then the external trigger is activated. The time delay in the response can be important and can lead to infections that can no longer be cured. Taking into account diverse coating applications, the detection methods should be portable, express, and have low equipment demand. In this regard, monitoring surface bacteria colonization through color changes seems to be an excellent possibility [213,214,215]. Most of the proposed works utilize the external changes in pH as a marker of bacterial strain development [216,217]. Such an approach is cheap and scalable, but it lacks reliability (because of the absence of bacteria-associated pH changes) and cannot indicate the initial stages of surface colonization (sufficient pH changes may occur only at a high degree of bacteria colonization). Thus, alternative approaches, based on the specific biomolecules’ appearance should be developed [218,219].

**Figure 21:**
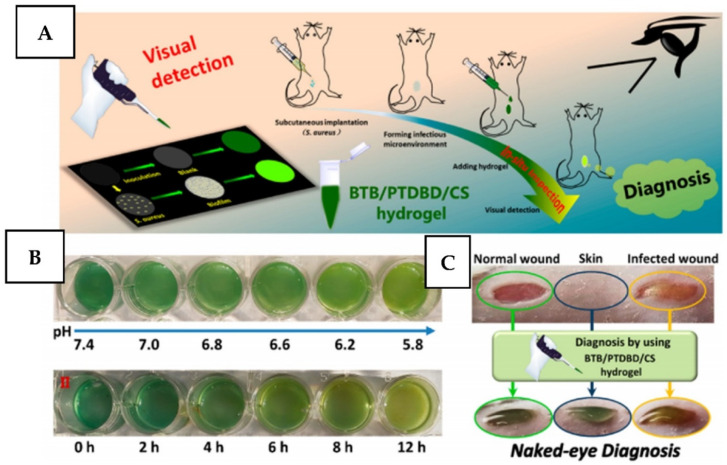
(**A**) A schematic illustration of visual detection of a bacterial biofilm and diagnosis of bacterial infection of mice by using bromothymol blue/(NIR)-absorbing conjugated polymer/chitosan (BTB/PTDBD/CS) hydrogel. (**B**) Detection of different pH (I) and growth of bacteria (II) by using the BTB/PTDBD/CS hydrogel. (**C**) Photographs of diagnosis of bacteria-infected wounds of mice. Reprinted with permission from ref. [222]. Copyright 2020 American Chemical Society.

The combination of detection and bacteria-killing was recently reported in [220,221,222], in which pH served as an indicator of bacterial development, signalizing the necessity of an external trigger—NIR irradiation. Similarly, in ref. [222], the authors used pH-sensitive dye thermosensitive hydrogels for pH-based diagnostic and photothermal treatment of the material surfaces (Figure 21).

Several works have also reported the combination of antimicrobial action (drug release) and simultaneous observation of bacteria-killing/survival [223,224]. Proposed approaches, based on optical changes and bacteria combating by nitric oxide or release of incorporated antimicrobial agents, which were not activated by an external stimulus, potentially provide back-response in the observation of bacteria-killing efficiency, which is an interesting opportunity for time-balanced application of coating triggering. Finally, the range of bio-inspired smart coatings should be mentioned. Such structures combine the morphology, copy the same objects from nature (which ensure the bio-repellency functionality), and additionally introduce externally activated antimicrobial agents [225,226,227].

It should be also noted that at present, the number of works reporting bacteria detection and on-demand antimicrobial activity activation is not so high and the above-mentioned problems should be addressed in the future. In particular, key questions, which should be solved are the introduction of faster and specific enzyme-based bacterial detection or expansion of the range of external stimuli, which can be applied for coating activation. Attention should be paid to the reliability of the received signal and the simplicity of observation because the well-known “signal” coatings, based on the pH-sensitive dye can provide a signal due to nonbacterial induced pH changes, in addition to continuous dye degradation and functionality loss. Signal receiving, based on enzyme-specific coating response could be more accurate, but it opens the questions of universality (detection of all dangerous strains) and cost. Therefore, the creation of sensitive and on-demand activated coatings should be performed by taking into account the targeted area of application, namely requirements for mass production, number, and a type of potentially represented bacterial strain(s), condition of coating utilization, including secondary factors as, for example, X-ray irradiation at customs control.

## 3. Outlook and Further Perspectives

We have reviewed the key approaches for the design of smart surfaces with a physically activated antimicrobial response (Table 1 gives a summary of the described approaches, including the common and well-known, as well as recently described advanced strategies). Such surfaces are extremely desired in the field of food packaging, wound dressing, implant protection, as well as the creation of safe surfaces on various hand-touched surfaces in public transport, or other social places. Of the main physical stimuli, temperature, light, electric, and magnetic fields as well as mechanical stress were discussed in detail. Such stimuli may induce antimicrobial surface protection through various mechanisms, among which the main attention was paid to the activation of drug release and changing of surface morphology with the related (bio)contaminant-removing performance. Most of the reviewed approaches can be used for combating the problem of bacterial surface contamination, but many issues related to the undesired release of drugs, coating passivation by bacteria residues, degradation of grafted antimicrobial agents, worsening of shaped surface features, as well as overall coating stability remain unsolved in the field of single-stimulus activated coating.

Besides the well-known physically activated coatings, we paid special attention to the design of attractive surface protection systems and materials, which can be activated by the simultaneous application of two stimuli, which can induce antimicrobial and antifouling actions, simultaneously induce bacteria-killing by several mechanisms or by combining the functions of surface colonization detection and on-demand activation of a coating. In this field, additional and significant progress can be expected soon because such approaches can enable better control of coating behavior, activation of antimicrobial properties at the right place at the right time, and prevent the bio-related passivation or development of resistant bacterial strains. It should also be noted that the majority of multifunctional coatings are based on relatively sophisticated materials and their industrial scaling can be questionable. Moreover, the simplification of multifunctionality realization without deterioration of overall bactericidal properties should be solved.

Additional questions, which should be mentioned for both single or multifunctional smart coatings are related to the development of methods for their stable deposition, potential environmental toxicity, safe and disposal utilization. Thus, many remaining and essential issues, attributed to the further development of economically feasible and scalable multifunctional coatings should be solved in near future.

## Figures and Tables

**Table 1:** Common methods of antimicrobial surface external activation by a physical stimulus and several recent more advanced approaches.

**Simple Methods of Surface Antimicrobial Response Activation**	
**Stimuli**	**Mechanism of Actions**
Temperature	Temperature-governed antimicrobial releasePhase transformation of grafted polymers and repellence of attached bacteria or biomolecules
Light	ROS productionLight-to heat conversion and bacteria-killing
Electric field	Reversible antimicrobial entrapping/releaseExternal control of surface morphology and wettability (including slippery and bio-repellency)
Magnetic field	Remote triggering of drug releaseSurface shape control—mechanical killing
Mechanical forces	Mechano-induced antimicrobial agent(s) local extrusion
**More Sophisticated Approaches and Recent Trends**	
**Approach**	**Advantages**
Double and multi-stimuli control	Better control of antimicrobial agents release and activation; simultaneous utilization of several surface protection mechanisms
Killing and release	Prevention of surface deactivation due to killed bacteria residue or their metabolic products
Detect and kill	Ability to apply external physical triggering in the right place at the right time

## Data Availability

Not applicable.

## Abbreviations

AFM	Atomic force microscopy
EF	Electric field
FITC	Fluorescein isothiocyanate
GLM	Gallium-based liquid metal
MRSA	Methicilin-resistant *Staphylococcus aureus*
NMP	Nitroxide-mediated polymerization
LCST	Lower critical solubility temperature
LED	Light-emitting diode
PEG	Polyethylene glycol
PMMA	Poly(methyl methacrylate)
PNIPAm	Poly(*N*-isopropylacrylamide)
PVDF	Polyvinylidene fluoride
QAS	Quaternary ammonium salts
RAFT	Reversible addition−fragmentation chain-transfer polymerization
ROS	Reactive oxygen species
TA	Tannic acid
TPP	Tetraphenylporphine
